# Mobile Health (mHealth) Technology for Eating-Related Behaviour and Health Problems Related to Obesity in Japan: a Systematic Review

**DOI:** 10.1186/s13030-025-00331-1

**Published:** 2025-06-16

**Authors:** Kazuhiro Yoshiuchi, Anna Brytek-Matera

**Affiliations:** 1 Department of Psychosomatic Medicine, The University of Tokyo Hospital, Tokyo, Japan; 2 Department of Stress Sciences and Psychosomatic Medicine, Graduate School of Medicine, The University of Tokyo, Tokyo, Japan; 3 Institute of Psychology, University of Wroclaw, Wrocław, Poland

## Abstract

**Background:**

Over the past decade, mobile health (mHealth) technologies have been increasingly utilised to address eating behaviours and diet-related chronic diseases. Nevertheless, research assessing the momentary clinical characteristics of these conditions remains limited in Japan. This study provides an up-to-date overview of research using Ecological Momentary Assessment (EMA) technologies in Japanese clinical and non-clinical samples through a systematic review.

**Methods:**

We reviewed studies extracted from MEDLINE via PubMed, Web of Science Core Collection and Scopus databases, spanning the period from 2004 to 2024. Our systematic review followed the “gold standard” Preferred Reporting Items for Systematic Reviews and Meta-Analyses (PRISMA) guidelines. Additionally, the Checklist for Reporting EMA Studies (CREMAS) was used to evaluate the quality of included studies.

**Results:**

From an initial pool of 32 potential articles, 4 studies met the inclusion criteria. A total of 75% of the studies were identified as using Personal Digital Assistants (PDAs) as the primary mHealth technology for EMA data collection.

**Conclusion:**

Available evidence suggests that the use of a PDA-based approach may help improve the management of eating behaviour and monitoring diet-related chronic conditions in the Japanese population. Furthermore, integrating EMA into clinical practice could enhance the effectiveness of existing treatments and support ongoing health monitoring.

## Introduction

Japan exhibits a notably low prevalence of obesity compared to Western countries. The age-standardised prevalence of obesity among adults (18 + years) in Japan was 3.7% in women and 4.8% in men in 2016 [1]. These rates were significantly lower than those in the United States, where obesity affected 37% of women and 35.5% of men and was the highest among all WHO regions [1]. In 2019, the prevalence of overweight and obesity among OECD countries was lowest in Japan at 27.2% (for comparison, Mexico had the highest rate, exceeding 75%) [2]. The recent study [3] has demonstrated the stable annual estimates of mean body mass index (BMI) among adults across the 47 prefectures of Japan over four decades (from 1975 to 2018). In addition, the prevalence of obesity has remained considerably stable among women over the past three decades [4]. In contrast, the incidence of overweight and obesity has approximately doubled in men compared to women. Notably, the most significant change has been observed among men in their 20 s, whose obesity levels have nearly doubled [4].

The Japan Society for the Study of Obesity [5] defines ‘obesity disease’ as a body mass index (BMI) of ≥ 25 kg/m^2^, which is lower than the World Health Organization [6] classification that considers a BMI of ≥ 30 kg/m^2^ as indicative of obesity. This discrepancy reflects regional differences in body composition and health risk assessments, with JASSO adopting a more sensitive threshold to identify individuals at increased risk of obesity-related health conditions within the Japanese population. Japanese individuals face a higher risk of obesity-related health complications compared to non-Asian populations [7]. For example, they tend to experience health risks associated with excess weight at lower BMI levels than Western populations. Additionally, at the same BMI level, Japanese individuals often have a higher proportion of body fat, particularly greater visceral fat accumulation in the abdominal region. This increased visceral adiposity can contribute to a heightened risk of cardiovascular disease and glucose intolerance among Japanese individuals compared to Western populations with similar BMI levels [8]. Given the above, excess weight remains a significant focus of public health policy in Japan, driven by concern about its potential impact on Japanese society and health outcomes [7].

One effective approach to addressing the rising prevalence of obesity is the promotion of a healthy, balanced diet. This approach not only helps in weight management but also plays a crucial role in preventing diet-related non-communicable diseases such as diabetes, stroke, heart disease, and cancer [9]. The Japanese diet, along with the Mediterranean diet, is highlighted as an example of a sustainable and healthy diet. The typical Japanese diet is characterised by a high intake of plant-based foods (such as soybeans) and fish (specifically n-3 polyunsaturated fatty acids) and and nonsugar-sweetened beverages (such as green tea), complemented by a more modest Westernised component that includes meat, milk, and dairy products [1]. This dietary pattern is believed to contribute to Japan’s relatively low obesity rates and overall good health [1]. However, recent years have seen an increase in red meat consumption, and diet-related non-communicable diseases remain the leading cause of mortality [10]. Therefore, addressing eating behaviour and health problems related to obesity is crucial for maintaining public health and preventing the rise of obesity and related health issues.

### Ecological momentary assessment and mobile health (mHealth) technology

Ecological Momentary Assessment (EMA) [11] facilitates the study of lifestyle experiences in real-time and in natural settings. This methodology uses monitoring or sampling strategies to assess phenomena as they occur in everyday contexts over time. The term ‘Ecological’ emphasises data collection within the participants'natural environments, ensuring observations reflect real-world contexts. ‘Momentary’ indicates that assessments focus on participants'current feelings and behaviours, capturing immediate experiences. ‘Assessment’ signifies that multiple evaluations are conducted across different moments, allowing for the development of a comprehensive and dynamic profile of behaviour [12]. To summarise, these approaches facilitate real-time data collection in natural environments, thereby enhancing the accuracy and ecological validity of the findings. Many of the limitations associated with traditional data collection methods—such as the focus on nomothetic or between-person analyses, limited generalizability to real-world contexts, reliance on retrospective reports, and susceptibility to memory biases—are effectively addressed through EMA research [13].

Wearable devices such as smart rings, smartwatches, and fitness bands, along with personal digital assistants and smartphones, are prime examples of mobile systems designed for personalised healthcare monitoring. These technologies enable comprehensive, real-time health management, empowering users to track and respond to their health needs effectively [14]. They have gained significant prominence in the field of healthcare, particularly in addressing eating behaviours and the management of diet-related chronic diseases such as obesity, diabetes, and disordered eating. By capturing moment-to-moment data, mHealth solutions have the potential to provide a more nuanced understanding of the complex factors influencing eating patterns and psychological states, ultimately leading to more effective, individualized treatment strategies. Despite the promising advancements globally, research exploring the dynamic, real-time clinical characteristics of these conditions remains limited within the Japanese context. There is a paucity of studies utilising EMA techniques to capture these daily fluctuations in naturalistic settings. This study aims to address this gap by providing a comprehensive, up-to-date overview of existing research employing EMA technologies in Japan. We conducted a systematic review, identifying and analysing studies that used mHealth technology, precisely EMA methodologies, to measure eating-related behaviour and health problems related to obesity in Japan in both clinical samples, such as adult individuals diagnosed with diabetes, and non-clinical populations.

### Methodology

This systematic review of the literature was performed using the following databases: MEDLINE by PubMed, Core Collection of Web of Science by Web of Science and Scopus. The search comprised the period between 2004 and 2024. The search strategy followed the guidelines from Peer Review of Electronic Search Strategies (PRESS) [15]. A total of 4 keywords were adapted to each of the chosen databases: mHealth, eating, Ecological Momentary Assessment, Japan. The searching general syntax was: (“Ecological momentary assessment” OR “mHealth”) AND (“Eating”), AND (“Japan”). All search terms utilised combination searches to ensure all variations of a term were captured. Inclusion criteria for this review were as follows: (1) full-text articles published in English, (2) used EMA-based data collection method, and (3) focused on the assessment of eating-related behaviour and health problems related to obesity in Japan. No exclusion criteria were applied based on sex, age, or clinical status. Reviews, editorials, protocols, book chapters and studies published as conference abstracts were not included in our systematic review.

**Fig. 1:**
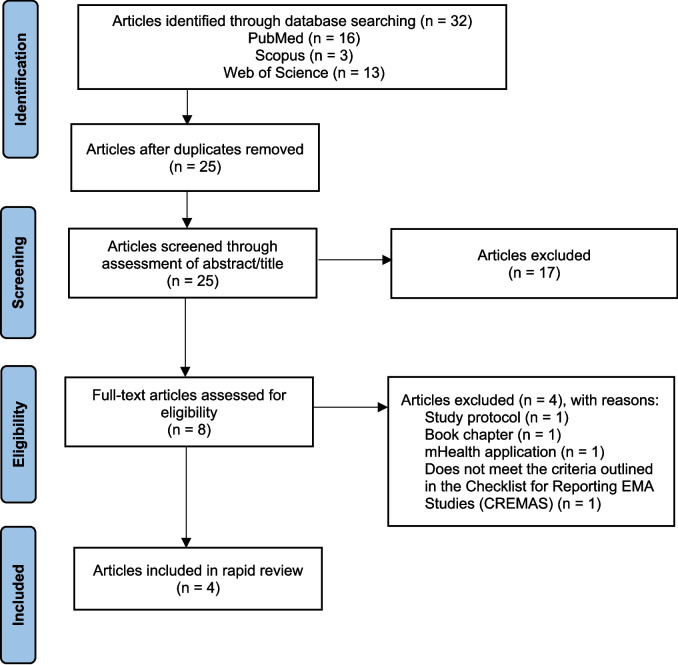
PRISMA flow diagram of search results

Our systematic review followed the “gold standard” Preferred Reporting Items for Systematic Reviews and Meta-Analysis (PRISMA) guideline [16] (Fig. 1). We also used the Checklist for Reporting EMA Studies (CREMAS) [17].

## Results

A total of 4 studies from 32 potential articles on the use of EMA in Japan were included in this systematic review. Their main objectives and results are presented in Table 1.

**Table 1:** The objectives and results of the studies employing EMA technologies in Japan that were included in the systematic review

Authors (years)	Objectives of the EMA studies	Results of the EMA studies
Kikuchi et al. (2015) [18]	Assessment of momentary appetite over 24 h in healthy adults	• Hunger/fullness, cravings, and total appetite before meals were positively associated with energy intake in daily settings • Hunger/fullness, carvings and total appetite were negatively associated with anxiety and depression. In addition, cravings were also negatively associated with stress in daily settings
Inada et al. (2016) [19]	Assessment of the changes in daily calorie intake, body weight and haemoglobin A1c after using the developed self-care system using PDA for 6 months in patients with type 2 diabetes	• Daily calorie intake, body weight, and HbA1c did not change significantly over the 6 months
Inada et al. (2019) [20]	Evaluation of a long-term, quantitative investigation of the influence of antecedent psychological factors on calorie intake in outpatients with type 2 diabetes, using computerised EMA over six months	Preceding psychological factors influence the calorie intake of outpatients with type 2 diabetes in daily settings • Preceding psychological stress was positively associated with calorie intake from snacks in daily settings • Preceding psychological stress, anxiety, and depressive mood were negatively associated with calorie intake from regular meals in daily settings
Shinozaki et al. (2024) [21]	Examination of the relationship between momentary overall diet quality of meals and meal type, eating companions, eating location and screen-based activities in healthy adults	Meal type, location, and eating companions were associated with meal quality in this population, with differences between women and men in daily settings • Compared to breakfast, lunch showed a lower meal quality among males, and dinner showed a higher meal quality among women and men in daily settings • Eating away from home for women and eating with someone for men was associated with better meal quality in daily settings • Younger age and current smoking were associated with lower meal quality in both sexes in daily settings

A total of 75% of the studies (*N* = 3) used mHealth technology, mainly a personal digital assistant, to collect EMA data. Those studies were limited by a relatively small sample size (*N* ≤ 20). Only one study (25%) was published recently [20], while the majority (75%) of these studies were published more than five years ago (between 2015 and 2019). All studies were conducted with adult participants: half included nine outpatients with type 2 diabetes [19, 20], while the other half comprised 242 healthy Japanese adults [18, 21]. The duration of each monitoring period was as follows: 4 days (1 study [21]), 7 days (1 study [18]), and 6 months (2 studies [19, 20]). In addition, two studies included from 3 [21] to 5 waves of data collection [18].

## Discussion

The systematic review highlights the current state of EMA research using mobile health technology among Japanese clinical and non-clinical populations, focusing on eating-related behaviour and health issues associated with obesity. It demonstrated that the majority of the Japanese studies relied on mHealth technology for data collection, predominantly utilising personal digital assistants. Personal digital assistants were introduced in the 1990 s as a pioneering tool for collecting EMA data [22]. However, given the rapid advancements in mobile technology in recent years, it would be beneficial for future studies to use sophisticated smartphone applications. Smartphones offer significant advantages, including enhanced capabilities, greater user convenience, and more versatile data collection options [23, 24]. Incorporating these modern tools into Japanese research would enhance data quality and increase participant engagement. Smartphone-based EMA methods now represent the forefront of real-time data collection, enabling more efficient, accurate, and ecologically valid research. By leveraging the embedded sensors within smartphones, such as GPS, Bluetooth, and pedometers, researchers can unobtrusively collect objective data about participants’ immediate environments, including their contextual factors. These sensors also enable the precise monitoring of behaviours, such as physical activity levels and social interactions, in real time. Moreover, the online format of smartphone-based EMA studies facilitates fully remote data collection, allowing participants to engage from any location without the need to attend in-person laboratory sessions. This flexibility not only enhances participant convenience and compliance but also broadens the scope for diverse and large-scale sampling, ultimately leading to more ecologically valid and comprehensive insights into daily behaviour and experiences [13]. Another promising mobile technology tool worth incorporating into Japanese studies is the smartwatch, which is currently playing a pivotal role in health care. Looking toward the future, it is anticipated that ring-type devices for continuous monitoring of various physiological parameters (such as heart rate, oxygen saturation, blood pressure, blood sugar, body temperature, dietary monitoring) will complement smartwatches, together forming the two main pillars of wearable technology in health care [14]. This combination has the potential to enhance personalised health tracking, improve data accuracy, and facilitate early detection and management of health conditions. Incorporating both devices into Japanese studies could significantly advance the development of comprehensive, real-time health monitoring systems tailored to individual needs.

In future studies conducted in Japan, there is a significant need for more comprehensive longitudinal data to better understand the trajectories of eating and weight-related behaviours over time. Such data would provide insights into causal relationships as well as the possibility of evaluating the long-term effectiveness of potential ecological momentary intervention (EMI). mHealth digital technologies may form powerful treatment methods in the mental health field in Japan, particularly concerning eating- and weight-related behaviours. Our systematic review indicates that a PDA-based approach has the potential to improve the assessment of eating behaviours and the monitoring of diet-related chronic conditions within the Japanese population. By enabling frequent and accurate data collection, mHealth technology can provide healthcare providers with timely insights into patients’ habits and behaviours, facilitating more tailored and effective interventions. In other words, using the mHealth digital tools could support mental health care in Japan, making it more proactive, data-driven, and patient-centred. Finally, integrating EMA with other digital health tools, such as artificial intelligence and machine learning algorithms, could enhance predictive capabilities and intervention efficacy. Ultimately, advancing this line of research holds promise for improving the prevention and treatment of health-related chronic diseases in Japan, fostering healthier behaviours, and reducing the burden of these conditions on individuals and the healthcare system.

## Data Availability

Not applicable.
